# Notes of an European Tour

**Published:** 1846-07

**Authors:** F. H. Hamilton

**Affiliations:** Professor of Surgery in Geneva Medical College. Naples, Italy


// BUFFALO MEDICAL JOURNAL
AND
MONTHLY REVIEW.

VOL. 2. JULY, 1846. NO. 2.

### ORIGINAL COMMUNICATIONS.

ART. I.—*Notes of an European Tour.* By F. H. HAMILTON, M. D., *Professor of Surgery in Geneva Medical College.*

NAPLES, Italy.

I should do injustice to the medical literature of Naples, if I should omit to mention that here the ashes and dried skins of many saints and martyrs work miracles among the sick, and bring honor and good cheer to the churches and refectories of the Holy Men. Let me, therefore, at once direct the suffering pilgrim who travels hitherward in search of health, whether his disease be the hip or the gout, green sickness or blue, to the church of Santa Pauolo, the blessed depository of two saints and fifty-two genuine martyrs! Now you are to understand that a mere martyr ranks only as an assistant surgeon, while a dead saint may be regarded as a surgeon in chief: if therefore you would experience prompt relief apply direct to one of the saints, and dont forget to leave a liberal fee. I am told it facilitates the cure wonderfully, and it seems very probable.

Go also to the church of Santa Gennaro, the patron saint of Naples, and within a gorgeous chapel, built at a cost of one million of ducats, which the people erected in gratitude to their patron and protector who had arrested the terrible plague of 1526, you will find the head and two vials of the blood of the martyred saint. The blood having been gathered up and carefully bottled immediately after his execution by a pious old woman; in which little bottles it has been sacredly kept, as every body knows, ever since. This blood is a miracle in itself, for when taken from the small silver tabernacle and held before the head of Santa Gennaro it immediately becomes liquid, although but the moment before it was as cold and clotted as the blood of any murdered sinner. It is arranged that this mysterious phenomenon shall occur three times a year, viz: in May, September, and December, and on these occasions it becomes a kind spiritual thermometer to indicate the degree of religious zeal existing among the citizens; for if on presentation of the blood to the saint it does not promptly liquify, the people, who are always assembled in great numbers to witness the miracle, are taught that it is because of their sins, and that some grievous calamity, such as a famine, the cholera, or a fatal eruption of the neighboring mountain is about to occur—when forthwith they begin to confess, howl and pray aloud, wringing their hands and tearing their hair, calling now upon Santa Gennaro, and now upon the whole calendar of saints not to delay the miracle. And when at length with a sudden motion of the hand—a "presto and pass," executed with a skill which the most expert magician might envy, the priest holds up the precious drops red and glowing as if reanimated by the living spirit of their beloved patron, the joy and enthusiasm of the congregation know no bounds. * * * *

Mineral springs, although they have less fame in Naples as curative agents, than relics, are nevertheless numerous and valuable. As I pass daily the sulphur spring of Santa Lucia, on the quay of the same name, I have often stopped with the throng to drink the water. It is not as strongly impregnated with sulphur as the waters of Avon in New-York, but it is much more pleasant from being pretty strongly impregnated with carbonic acid gas. At this season of the year it is much resorted to by the people, from a popular notion that it serves as a preventive of fevers, a belief to which the authority of Cerillo* the patriot and physician has given sanction. Cerillo was also a great advocate of the cold water practice during the existence of fevers, and especially in cases of malignant fevers, giving his patients at the rate of twelve quarts of snow water every twenty-four hours during eight or ten successive days. He also directed the surface of the body to be often bathed with the same.

The "Acqua Ferrata" at the foot of Pizzo Falcone, is, as the name indicates, chalybeate, and although not disagreeable to the taste, it has much less reputation than its neighbor the "Santa Lucia."

In the neighborhood of the city are several thermal springs containing various minerals, salts, alkalies, acids, &c., &c., and varying in temperature from 80° to 180° of Fahrenheit; among these are the several springs in the island of Ischia and at Castel-a-Mare, on the opposite side of the

*When in 1799 Cerillo was arraigned for having sustained the authority of Murat and the liberties of the people against the king, he was asked, "What is your profession?" "A physician," he replied. "What were you under the Republic?" "A representative of the people." "And now in my presence," said the conceited and arrogant minister of police. "In faccio a te un Eroe," (In your presence a Hero,) replied the undaunted Cerillo.—Lady Morgan's Italy.

bay; the springs of Pozzuoli; La Solfatara in the famous crater of the same name; the "Acqua Bagnoli," two miles west of Naples, with a temperature of 107° Fahrenheit; and the hot baths of Nero whose heat is insupportable, being as high as 180° Fahrenheit. A few only of these valuable mineral and thermal waters are arranged with proper conveniences for bathing. The Italians, as both their streets and persons sufficiently indicate, having a prevailing dislike to all lustrations.

With regard to the climate of Naples as adapted to invalids, permit me to say that I believe a wide and fatal error prevails. It is true that its skies are *generally* clear, and its air balmy and delightful. In the winter the thermometer seldom falls below 40° of Fahrenheit, (during last February it fell below 30°—but this was an unusual event,) nor in summer does it often range above 80° in the shade. Naples is not therefore subject to *extremes* of either heat or cold. But such changes as do occur are *frequent* and *peculiarly injurious*, at least to the pulmonic invalid. Most of the year, during the day, with remarkable regularity, a sea breeze comes in from the south and west, called the "sirocco," whch is exceedingly relaxing and enervating, and although it does not greatly raise the mercury, yet I am prepared to bear witness that no degree of heat has ever so prostrated me, convincing me, despite my previous convictions to the contrary, that from 11 o'clock, A. M. to about 3 P. M., I must court the shade and indulge in the most undisturbed idleness. Such is the custom of all Italians, whether poor or rich. At this time of day you may see every where, in the fields and by the road side, the shepherd sleeping under the shade of trees—the vine-dresser under his portable thatched hut—the beggar and the lazzarone under high walls and market sheds—the chirping lizard is alone awake and abroad, frolicking nimbly over the face of the sleeper, yet never at all disturbing his sleep. At first I pointed to these customs as evidence of the idle habits of the Italian people, and I thought by climbing Vesuvius at midday to show them the difference between an American and an Italian. The result of my enterprize has been, that I am completely subdued, and until I cross the Alps again, I shall not repeat the indiscretion.

But to the "sirocco" of the day succeeds the land breeze, or the "tramontane" at night; in the summer generally pleasant and refreshing, and therefore none the less dangerous, but often exceedingly damp and chilling during the months of February and March, and indeed so long as any considerable body of snow rests upon the Appenines. Even now in the beginning of June, we have had a *storm* from the North-east, which commencing at 4 P. M. has continued during the whole night, not a mere drizzling rain, but all this time the black clouds which hung over the city, were unloading themselves in torrents. I was walking in the "villa reale" when it commenced, and was most thoroughly drenched before I reached my lodgings. To-day I have symptoms of a cold for the first time since I left home. But with Neapolitan rains there is one redeeming feature—the soil—a porous tufa, drinks in the water like a sponge, and this morning the streets are as dry as before the rain.

These are the circumstances which, in my opinion, render Naples peculiarly unfitted for a pthisical patient. To the correctness of which opinion, the pthisical wards of the Hospital of Incurables—the small but crowded English Cemetery, on the beautiful summit of Posillipo, and the English physician, for many years a resident of Naples, all bear concurrent testimony.

It is said, however, that there is something especially serviceable to the dyspeptic and hypochondriac, in the highly electric condition of the atmosphere, (ascribed to the influence of Vesuvius.) It is possible—yet I am more disposed to ascribe the benefit derived by this class of patients, to the influence of enchanting scenery and to the great number of classic and exciting journeys which the sight-seeing traveller is compelled to make about Naples. * * * * * *

Having first visited and studied at the Museo Borbonico, the statuary, paintings, jewelry, bones, surgical apparel, &c. removed from Herculaneum and Pompeii, I was better prepared to examine the cities themselves. Those cities which are now attracting so large a share of the interest and attraction of Antiquarians, whose walls and foundations were laid before the Siege of Troy, (more than 3,000 years ago,) and of whose beauty, salubrity, opulence and maritime advantages, Strabo, Homer, Tatius, Pliny and Cicero have alike spoken, as well as of the "grandeur and magnificence of their public buildings and spectacles, and the talents and enterprize of their citizens:" and which, in the very noon-day of their prosperity, were suddenly, amid a scene of terrible sublimity to which the history of the earth can furnish no parallel, sealed up, as if to remain closed until the earth and the heavens should be dissolved together: for when the convulsions had ceased and the flood of lava, with burning stones, had cooled and settled over all that broad and beautiful valley, not a battlement or a tower stood up to tell where the walls and the temples once stood. But now, after nearly eighteen centuries, those buried cities, with very many of their old inhabitants, with their gold and silver and bronze ornaments about them, with their corn, and olives, and fruit, with their wine goblets and amphorae, with their last loaf stamped and prepared for the oven, and the fragments of their last meal scattered about them, all just as when the last fatal hour came upon them, are again brought to light. It is to me like a strange vision, revealing, as if time were nothing, the long past with the clearness and accuracy of the present.

With an intelligent Italian guide, I descended into the Via Domitiana, leading to the western gate of the city of Pompeii ; at the very commencement of which is stationed a small guard to watch carefully all who go in or out, and defend from plunder the sleeping city.

The first house we entered, was the beautiful villa of Arius Diomed, a Freedman of wealth, standing some distance without the gate. You have doubtless read the description of this house, given by Bulwer, in his "Last Days of Pompeii ;" and I need scarcely say, since he wrote on the spot, that it is sufficiently correct ; but I looked in vain for those ideal beings with which, after so many centuries, this inimitable novelist has again peopled it. We walked boldly into the open door-way, and through the large atrium, but no "Medon" came out to interrogate our rudeness, or to announce our presence to old "Diomed." The stone well was there, and the mark of the rope worn deep upon its curb, but no water or maid to draw. We trod through the silent and deserted chambers, the dressing rooms, sleeping apartments, library, baths, kitchen, and passing into the garden, "yonder," said the guide, "close by a postern, which once opened upon the sea lay, when this house was first opened, the skeletons of Diomed and his servant, who, with their portable treasures, were attempting to escape, when the storm overtook them.

Underneath are the vaults, a long, narrow, subterranean cellar, lighted only by small loop-holes which opened on a level with the surface of the garden. Twenty human beings here perished, of whom two were children, and one a new-born infant. A large amount of female, gold, bronze and silver ornaments lay scattered about, and along the wall are the amphorae or wine jars, broken in at the top and filled with ashes, but which have never been disturbed. All of the bodies were huddled towards the door, as if in the last struggles for life they had sought again to escape from their narrow, suffocating prison.

Passing by many other places and buildings of scarcely less interest, we enter the city by the Herculanean or Western Gate, and the first house on the right, is the Post House of Albinus (as the name over the door, and the *chequers* upon the wall indicate.) I notice this house, because upon one of the pilasters is sculptured a "talisman." For I wish to convince you that people of that remote age, were well nigh as credulous as men of the present. But they were nevertheless more consistent in this, that they were not forever dropping old follies for new.

A belief in the "mal ochia" or "evil eye," is as old as the days of Homer, among the Greeks; and the Greeks in their migrations into Italy, had brought it with them. The belief was, that some men, and especially women, had the power, by turning their eyes upon any person or house, to bring a curse upon them, unless the person or thing so looked upon was protected by a talisman. 'And even now, I have seen many shops and stands in Naples, where talisman's of various forms are sold, as protections against the "mal ochia," and as preventives of various maladies. The same superstition with regard to the evil eye, also, I am told, prevails in many parts of Ireland.

Nearly opposite is the "Surgeon's House" where were found forty surgical instruments, and other paraphernalia of a surgeons office. The instruments I have examined at the Museo Borbonico, and they consist of clumsy forceps, scalpels, a trocar, cauterizing irons, catheters, &c., &c. The cauterizing irons are altogether the most numerous, and in themselves give us a pretty fair idea of one feature of ancient surgery, the cautery being almost a universal panacea for surgical diseases. It was on account of the constant use of these instruments that Archagathus, the Greek surgeon was driven by the enraged populace from Rome; they declaring that he was an "executioner" rather than a "healer of wounds." The catheters however, are perhaps the most intersting memorials, since some of them being straight, fully prove that the use of the straight instrument is not as claimed, a modern suggestion. The use of a catheter in some form dates from Erasistratus, the Alexandrian physician, about 300 years B. C. The catheters are made of bronze, and have a fenestrum, like a modern instrument. The forceps, cauteries, &c. are of iron, and are much rusted. I enquired for the speculum vaginæ, which is said to resemble, almost precisely, "Weis's dilator," but with one handle made of ivory and one of bronze: the cicerone knew nothing of it.

These reliques were sufficiently interesting, as illustrations of Pompeian surgery; but I was of all most curious to know how my fellow-tradesman had lived in these olden times; and I have been gratified, and withal, not a little surprised, to find that he had occupied one of the best houses in Pompeii. And, as it is a fair specimen of the private dwellings of the better class of Pompeians, I will describe it briefly. It stands directly on the narrow side-walk of the Via Domitiana, and is built of small blocks of lava and thin brick, faced with stucco. It was certainly two stories high, possibly three, but the flat roof and the upper part of the walls having fallen, it is impossible now to determine what was the original height. Only two or three private buildings in Pompeii have been found to have had three stories. The front is about sixty feet and the building extends through to the next street. Entering the large front door, we passed through a hall called the prothyrum, into an open court, (the atrium,) nearly square, and having in the centre a square impluvium or cistern for rain water, without a cover, and very shallow. On two sides of the atrium are small rooms or alcoves, like the cells of a prison, with a door opening into the atrium, and containing a stone platform elevated a few inches above the mosaic pavement, designed for the beds. These rooms are the cubicula or bed-rooms. In the rear, communicating by a door with the atrium, are other small and more private apartments for the ladies, also the kitchen, &c. The front lower rooms were occupied as shops. You will not now be surprised at the appearance of comfort and even luxury which this house exhibits, when I tell you that upon several of the leaden weights found here was the following inscription :—" *They who pay will be served.*"

Some distance farther on is a druggist's shop, with a serpent eating the pine apple, as a sign. The serpent being the Æsculapian emblem of wisdom, and the pine apple being an emblem of death; the whole indicates, therefore, the power of medicine to vanquish death. A small box of pills are among the treasures seen at the museum, whether taken from this house or another, I cannot say.

Still walking along the Via Domitiana, past the Trivii, and turning to the left, we came at length to the Public Baths, occupying nearly the whole of an irregular square. Of all the buildings in Pompeii, these are in the most perfect condition, and perhaps they are the most perfect of any similar remains found in Italy, yet by no means as extensive or magnificent as the imperial thermæ of Rome. Their comparatively limited extent, however, being, as it were, a compendium of the baths of Caracella, will render their description the more valuable, since you can thus obtain at once, a clear conception of the style and plan of those wonderful monuments which contributed alike to the cleanliness, luxury and hygiene of the ancients.

Turning into the beautiful street of Fortune, to gain the only entrance now left open, we stepped from the narrow paved foot-path into a stone door-way, whence through a covered vestibule we came to an open court, called the atrium; a space about sixty feet square, upon two sides of which are the handsome doric columns of a portico whose roof is gone; the third side exhibits portions of heavy arches, and the fourth is a plain white wall. Along the wall, under the porticoes, are low stone benches, and on the north side is a small recess or ala.

Here, then, in this court, the visiters of the thermæ were accustomed to wait their turn, for probably not more than thirty could be accommodated at one time, and since it cost only one quadrans, (about half a cent,) doubtless the crowd of expectants was often considerable. They walked hurriedly, therefore, back and forwards, across the atrium, as Celsus advised his patients before they entered the baths—or, if merely in pursuit of a luxury, they stood here and there under the shade of the arches and collonades, or perhaps loitered away to the south wall, whose smooth white surface served as the public album or place of advertisements—the only substitute which the Pompeians had for our placards and cheap newspaper notices. These advertisements were written with red chalk or paint, and one of the notices read as follows:

DEDICATIONE POLY—MAIO PRINCIPI COLONIÆ
FELICITER .... RIVM . MVNERIS . CN . ALLEI . NIGER......
SPARSIONES . VELA . ERVNT . VENATIO ATHLETÆ

Which the learned have translated—"On occasion of the dedication of the baths, at the expense of Cnæus Alleius Nigidius Maius, there will be the chase of wild beasts, athletic contests, sprinkling of perfumes and an awning—Prosperity to Maius, Chief of the Colony." A similar announcement was made upon one of the arches of the western gate as you enter the city, and they indicate that the baths were new and had not yet been dedicated. It was on this very occasion the people were gathered at the amphitheatre, when at a little past noon, on the 24th of August, A. D. 79, the fatal eruption burst upon them.

The recess on the north side of the atrium, was doubtless occupied as a ticket office by the balneator or keeper—where he received the quadrans and gave out the tickets; behind him was a small hole through which he might see whether the baths were full, and in which sat his lamp protected by a crystal shaped glass—the smoke has sadly discolored the ceiling. The poor fellow himself escaped from the building, but he left behind his ivory handled sword and its leathern sheath.

From the north east angle of the court we passed, as the bathers were wont to do, through a vaulted coridor into the apodyterium. The ceiling of the corridor was painted in blue and studded with stars. In this passage were found about five hundred small lamps in terra cotta, used for lighting the different portions of the building, and as many more were found in other apartments.

The apodyterium, or undressing room, now partly fallen, is a large hall, with a high arched ceiling—the ceiling painted in yellow panels with crimson borders—the walls were yellow and the cornices when first opened was curiously colored with various grotesque devices, which are now quite obliterated. Extending around most of the apartment, is a low platform, surmounted by stone seats, and over the seats is a range of holes, where pegs are supposed to have been placed, to suspend garments. Only one window exists in that part of the ceiling which remains, and this was glazed with plate glass, ground upon one side, and set in a copper frame. Similar glass lights have been found in other parts of the thermæ.

The temperature of this room was like that of any other room in this climate having solid stone walls, and receiving but a dim light; it was cool and refreshing, and was therefore a place where the visiters often loitered some time before they began the regular course of thermal discipline.

At the southern extremity of the apodyterium, communicating by a door, is the bijou of the thermæ; a circular room, lighted by a skylight in a handsome circular dome, which springs from an ornamented cornice; occupying the centre of the room is a white marble cistern, circular also, twelve feet ten inches in diameter, and about three feet deep, with a marble step about one foot below the lip of the basin; a marble footpath also encircles the whole. A brazen pipe projecting through the wall, threw the water across the ambulatory into the bath.

On four opposite sides of the room, are vaulted alcoves, with red concas and blue walls. The walls of the entire apartment were yellow, relieved with green branches, and the dome was blue.

Thus I have described to you the cold bath of Pompeii, the most perfect specimen of the ancient natatio, as it was called, now known to exist. Many bathers never went beyond this room, despising the more luxurious and effeminate recreation of the tepidarium and calidarium, and this was especially becoming a "fashion" about the time of the destruction of Pompeii. The custom having been introduced by Augustus at Rome, and having gradually extended thence, throughout the Roman dominions. Galen however taught that this bath should only be resorted to by the healthy, temperate and fleshy; being altogether too depressing for the more feeble. Generally the natatio, although adjoining the first hall, was used in returning, as a final lustration.

You shall accompany us now from the apodyterium, through a door so suspended on leaning hinges, that it shut by its own weight, into the tepidarium, or, as I shall call it, the dry hot air room, for it was warmed by a large portable brazier, somewhat like those smaller bracciere which may now be seen in the houses of modern Italy, and called by the Latins a foculare. The brazier is of bronze lined with sheets of iron and floored with a brass grate, to support the hot brick, pumice and charcoal. It is seven feet long, by two and a half wide, and is ornamented along its rim by dwarf battlements, and lotus leaves, and a small figure of an ox in alto-relievo. There are also three bronze seats. each about six feet long, the supports representing the head, legs and feet of an ox. The name of Marcus N. Vaccula is inscribed upon the seats.

The ceiling of the north end of this apartment has fallen, but enough remains to give an idea of its exquisite beauty, being wrought into various panneled copartments, and worked up with a multitude of figures and delicate corinthian tracery, painted in glowing colors. But the most striking feature of this apartment is the colonade of atlantes, of which thirty or forty remain, standing upon a cornice against the wall a few feet above the floor and supporting upon their heads and uplifted arms a richly carved entablature, which extends around the whole room. These atlantes are short ugly looking figures, carved in stone, with grizzly beards and grotesque faces and entirely nude, except a small tunic below the waist; in short they are but miniature representations of the hideous wooden giants, Gog and Magog, to be seen in the old Guildhall at London. The niches between them were used as shelves, where the bathers deposited their unguents, essences lamps, &c.

In the tepidárium, were practiced the luxurious operations of inunction, strigillation, friction, kneading, powdering, &c. &c. Sometimes preceding and at other times succeeding the use of the hot bath, in the adjoining laconicum.

The laconicum, or third principal apartment, was entered by a door suspended in the same manner with that between the undressing room and the tepidarium, it was also additionally protected by a curtain, suspended over the door; the rings upon which the curtain hung have alone been preserved. The laconicum is thirty seven feet long, by seventeen wide: its ceiling is vaulted, and groined but plain: the marble pavement is suspended, admitting hot air from the furnace, through its open copartments, which copartments or flues extend, also into the lateral walls. At one extremity of the laconicum is the calidarium, or hot water bath. It is a marble cistern two feet deep, fifteen long and four wide, with a long step halfway down from the lip of the cistern, upon which the bather might sit: to enable the body to lay at length in the water without submerging the head, the floor of the bath has a considerable slope. The hot water was admitted by a pipe, directly from the cauldron, in an adjoining chamber.

At the opposite extremity of the laconicum, stood a vase of solid marble, resting upon a short thick shaft of tufa. The vase is eight feet in diameter and very shallow, having in the centre a projecting tube, from which arose a *jet d'eau.* This was the labrum in which after taking the hot bath, the visiter sat to receive upon his head a light shower of cool water, before he concluded his lavations. Some however preferred returning to the natatio as we have before intimated, for says Galen, "who neglects the cold bath at the door, is in danger from open pores, on returning into the open air". The same practice is now pursued in the eastern hamem, and also in many of the more northern countries of Europe, where as in Russia, in the midst of winter they plunge from the hot bath into the snow banks, or into the nearest stream of cold water; a practice which is rendered the more safe from the high degree of animal heat, and excitement and consequent power of resistance to cold which they have obtained from immersion in the calidarium.

Separated by a small room containing the furnaces, from the male apartments, and by a hall, are the female apartments, which are neither as large, as richly ornamented, or as convenient as those we have now described: the ladies only visiting the baths for purposes of health or cleanliness, and not as a mere luxury, as was the case with the men, some of whom spent the greater part of every day in the public thermæ. Beside this, the ladies, at least those of rank or wealth, had their own private baths at home, as may be seen in many of the houses at Pompeii, and probably none but the poorer class of females resorted to the public baths.

They consist of three principal rooms, with vaulted ceilings, and skylights, and contain a ruined labrum, a natatio, &c. The floors are of tessellated marble, and the walls pannelled and variously colored.

I forgot to tell you that in the laconicum of the male thermæ, I enjoyed a luxury of which few epicures can boast. It was a bottle of ripe Pompeian wine, which the present balneator (for those apartments are occupied by a keeper) drew out, in my presence, from underneath the suspended pavement, and which he gravely assured me was poured from one of the amphoræ discovered in the vaults of Diomed. Those that I had seen were broken and filled with ashes; but to doubt the word of a relique keeper is a kind of absurd infidelity of which I hope never to be found guilty. So never talk to me again of "old wines" when I have uncorked that which has seen 18 centuries, or of wine which has made the voyage of the Indies, ween I have tasted that which has been rocked and warmed at the foot of Vesuvius since the days of Titus.

Lest I may become tedious, I shall omit even to enumerate the thousand other things of interest found both in Pompeii and Herculanean—the many temples also, the theatres and amphitheatres, and other public buildings that have been laid open, among all of which none is more perfect than the beautiful temple of Isis. Isis I believe held in Egyptian mythology the same relation to the science of medicine as did Apollo in the Grecian, being worshipped on the Nile as the goddess of medicine. Diodorus Siculus says of her, that while a mortal, she invented many excellent medicines, and hence, since her reception among the deities she is applied to for the cure of distempers, indicating the remedies often by dreams. Galen mentions several plasters which bear her name.

From an inscription, it appears that this temple had been thrown down by the earthquake of 63 and rebuilt by Celsinus. For although her worship had been introduced from Egypt, she had become latterly the favorite and fashionable goddess, and her oracles, always the most ambiguous, were deemed the most infallible of all the deities.

I considered myself lucky, therefore, in being able, upon certain matters which weighed doubtingly upon my mind, to consult so venerable and faithful an oracle.

For lack of a priest, I directed my guide to enter the penetralia, and ascend the winding, and secret staircase, by which so often the lying priests had stolen behind the statue of their goddess; and which is now laid open to attest their impostures. And although the statue is gone from its pedestal, yet the most essential part, the opening through which the oracles were uttered, remains; and I proceeded. with due form, to demand of the goddess, whether my two wishes, then dominant, would be granted. To-morrow, a fair day to ascend the mountain—and, in due time, a safe and pleasant voyage home. In a clear, but solemn voice, the oracle gave response—" si senore."

My guide performed his part well, except in the suffix "senore," having in this forgotten, that, as a priest of Isis, he should assume the dignity of a god, rather than the servility of an Italian valet. "Well," I say, for the predictions were verified.

Within the "penetralia" were found two human skulls, the Isiac tables, covered with curious hieroglyphics, probably Egyptian, a box containing the sacred paraphernalia, &c.

In an eating room, belonging to the temple, was the body of a man; and a table, upon and about which, were eating utensils, egg shells, bread, bones of fishes, bones of a chicken, and also portions of a ham, which latter, the priests of Isis were strictly forbidden to eat. A religious ordinance which may be supposed to have been of Hebrew origin. With this forbidden repast, probably, the holy man was secretly refreshing himself, immediately after a public sacrifice, for in the adjoining court was an altar, upon which lay cinders and calcined bones, and beside it, a garland, used to crown the victims. Like the fat old friars of yore, he felt bound, occasionally, to relax the severity of his discipline, and to acknowledge, in secret, the hand of a liberal providence in all his creatures.

Another priest was found leaning against the wall of the kitchen, and a large sacrificial axe lay beside him resembling a modern broad-axe. With this, (as the broken doors themselves show,) he had hewn his way through two doors, and had made a strong impression upon the third, (the marks upon which correspond exactly with the blade of the axe.) The impressions were deep and repeated. The frame of the priest was large and bony, and in this last struggle for air, his powerful arm had sent home the blows fast and heavy, until exhausted with the fruitless effort, he sank suffocated and nerveless against the wall.

Many others were found in and about the temple. One, loaded with such treasures as were most portable, had fled as far as the Tragic Theatre. He carried with him more than 400 coins wrapped in a linen cloth.

I designed, when I sat down to these sheets, to subjoin, at the close, a description of Herculaneum, which is also partly excavated, yet not to the same extent with Pompeii—the former being buried to a depth of 70 feet below the surface. So that the work of excavation has been necessarily extremely slow; and, indeed, it is now entirely arrested. The inhabitants of the towns of Portici and Resina, situated directly above, becoming alarmed, lest the intermediate strata should give way beneath them.

A large amphitheatre, and a few private dwellings only, have been laid open. And as the traveller descends the long, deep shaft, which leads to these subterranean dwellings, with swarthy guides, and blazing torches, he is almost persuaded that he is entering the dark avernus of the ancient poets. Nor is the delusion abated, when he hears, (coming and going, at short intervals,) first, a low rumbling, as of distant thunder; then gradually becoming louder and heavier as the sounds approach nearer, until finally it vibrates along the arches of these hollow caverns, with a startling and deafening power.

But not only the carriages rattling over the rough stone pavement above, but even our own voices gave back a strange and hoarse echo from the distant chambers. By the light of the flambeaus our faces bore a livid aspect—the air was cold, damp and mephitic—and, chilled to the very bones, I was glad, after a rapid inspection, to hasten from this dismal place, to the warm, cheerful light of this upper world.

